# Putative epigenetic regulation mechanisms related to production, carcass and beef quality traits in Nelore cattle

**DOI:** 10.3389/fgene.2025.1593444

**Published:** 2025-06-27

**Authors:** Juliana Afonso, Woo Jun Shim, Tainã Figueiredo Cardoso, Jennifer Jéssica Bruscadin, Andressa Oliveira de Lima, Wellison Jarles da Silva Diniz, Bárbara Silva-Vignato, Ingrid Soares Garcia, Wei Liang Andre Tan, Priscila Silva Neubern de Oliveira, Aline Silva Mello Cesar, Gerson Barreto Mourão, Adhemar Zerlotini, Luiz Lehmann Coutinho, Marina Rufino Salinas Fortes, Luciana Correia de Almeida Regitano

**Affiliations:** ^1^ Animal Biotechnology Group, Embrapa Pecuária Sudeste, São Carlos, São Paulo, Brazil; ^2^ School of Chemistry and Molecular Biosciences, The University of Queensland, Brisbane, QLD, Australia; ^3^ Institute for Molecular Bioscience, The University of Queensland, Brisbane, QLD, Australia; ^4^ Post-graduation Program of Evolutionary Genetics and Molecular Biology, Federal University of São Carlos, São Carlos, São Paulo, Brazil; ^5^ Division of Medical Genetics, Department of Genome Sciences, Department of Medicine, University of Washington, Seatle, WA, United States; ^6^ Department of Animal Sciences, Auburn University, Auburn, AL, United States; ^7^ Department of Food Science and Technology (ESALQ), University of São Paulo, Piracicaba, Brazil; ^8^ Department of Animal Science (ESALQ), University of São Paulo, Piracicaba, Brazil; ^9^ Bioinformatic Multi-User Laboratory, Embrapa Informática Agropecuária, Campinas, São Paulo, Brazil

**Keywords:** *Bos indicus*, histone modification, H3K27me3, epigenetic, muscle

## Abstract

**Introduction:**

Understanding regulatory mechanisms like epigenetics can help improve beef production, carcass, and meat quality. Epigenetic states are dynamic and shaped by the environment, but due to limited studies and costly detection methods, alternative approaches are needed.

**Objective:**

Our aim was to identify candidate regulators linked to production, carcass and beef quality traits by describing genes putatively regulated by epigenetic mechanisms in the muscle of Nelore cattle.

**Methods:**

We *in-silico* identified discordantly regulated genes (DRGs) with the TRIAGE method and rank product analysis, using gene expression. We investigated the DRGs for being known bovine transcription factors (TFs) or co-factors (TcoFs) and tested the association of SNPs harbouring the DRGs with the traits. Using public muscle ATAC-Seq and ChIP-Seq data, we found that the associated SNPs were harboured in open chromatin sections of the genome and/or on histone modification regions.

**Results:**

We identified 51 DRGs across the traits and provided evidence of their regulatory status. 26 DRGs are known bovine TFs. A SNP upstream of the PITX2 DRG was associated with conjugated linoleic acid (CLA), 35 SNPs within or around the BTNL9 DRG were associated with backfat thickness (BFT) and 13 of the DRGs showed a regulatory impact over at least one trait.

**Discussion:**

The correlations identified among DRGs, differentially expressed genes and traits showed intricate relationships with various TFs and TcoFs, revealing the putative relationships of these elements with the traits. The *LBX1* and *HOXC10* genes are candidates with evidence to be regulators of the traits, while also being subjected to epigenetic regulation.

## 1 Introduction

The quest to improve quality and production traits in livestock has long been a focal point in agricultural research. Several factors influence these traits, including epigenetic regulation. Epigenetic states are reversible and can be affected by environmental factors (Jaenisch and Bird, 2003), being associated with specific phenotypes. One example of this association are changes in the cell phenotype that cause tumor cells in humans to grow and escape immune system attacks (Perrier et al., 2020). DNA methylation in the core promoter region of the *SIX1* gene in muscle tissues may be regulated by histone H4 and the transcription factor *E2F2*, impacting muscle development in Qinchuan cattle (Wei et al., 2018). Similarly, genome-wide differentially methylated regions and genes between obese and lean pigs underpin the role of methylation in lipogenesis (Yang et al., 2016).

Epigenetic mechanisms can also interact with single nucleotide polymorphisms (SNPs). SNPs associated with predisposition to autoimmune disorders in humans are clustered in genomic regions with epigenetic modifications of active enhancers in T or B lymphocytes (Liotti et al., 2022). In patients with Tetralogy of Fallot, a congenital heart defect, the histone modification H3K18ac binds to the promoter region of the *Cx43* gene, controlling its expression. A SNP (rs2071166) in the *Cx43* promoter region affects the binding of H3K18ac, influencing its expression and impacting the likelihood of Tetralogy of Fallot presentation. In cattle, unique SNPs likely to be causal variants for gene expression according to eQTLs analysis were associated with histone modifications in the mammary gland (Prowse-Wilkins et al., 2022), suggesting a link between SNPs, epigenetic regulation, and livestock traits.

Methods that detect epigenetic mechanisms, such as the TRIAGE method (Shim et al., 2020), can be useful since histone modifications represent one of the most conserved epigenetic mechanisms in animals (Tollefsbol, 2023). Combining the TRIAGE method with rank product analysis, as employed in our previous research (Afonso et al., 2020), can facilitate the comparison of contrasting sample groups which aids in the identification of regulators for each trait. The TRIAGE method identifies genes with a high probability of being epigenetically regulated and a gene expression regulator in a given sample, by ranking genes based on a multiplication of a repressive tendency score and their expression values. The repressive tendency score was given for each human gene by the TRIAGE’s authors. To compare the gene ranks between contrasting sample groups, we can use the rank product analysis.

Our hypothesis is that there are differences in epigenetic regulation between contrasting sample groups for a given trait, and that SNPs in or around the involved genes are also associated with the same traits. Therefore, our main goal was to identify candidate genetic regulators linked to production, carcass and beef quality traits through the investigation of epigenetic mechanisms in Nelore cattle.

## 2 Methods

### 2.1 Ethics declaration

All methods and experimental procedures employed in this study received approval from the Ethical Committee of Embrapa Pecuária Sudeste (São Carlos, São Paulo, Brazil, CEUA 01/2013).

### 2.2 Samples

The complete experimental population from which different subsets were used for this research encompasses 460 Nelore steers used previously in other research projects from our group, since 2013 (Tizioto et al., 2013). The animals were sired by 32 unrelated Nelore bulls representing the main genealogies of the time in Brazil and were born in three breeding seasons in the years 2007, 2008 and 2009, by artificial insemination. The animals were born in five different locations, being the Embrapa Pecuaria Sudeste (São Carlos, São Paulo, Brazil), Embrapa Gado de Corte (Campo Grande, Mato Grosso do Sul, Brazil) and in three private properties in the Mato Grosso do Sul and Mato Grosso states, in Brazil.

After rearing, between 2009 and 2011, the animals were housed in feedlots at Embrapa Pecuária Sudeste (São Carlos, São Paulo, Brazil) with *ad libitum* access to water and feed, as previously reported (Tizioto et al., 2013). At an average age of 25 months, the animals were slaughtered in a commercial slaughterhouse in Bariri, a city of the São Paulo state, in Brazil in accord to the protocols of the Ministério da Agricultura, Pecuária e do abastecimento (MAPA) and supervised by the Serviço de Inspeção Federal (SIF), both from the Brazilian government. The desensitization was done with a pneumatic penetration pistol. After slaughter, samples from the *Longissimus thoracis* muscle, located between the 12th and 13th ribs were collected. The harvested tissue was promptly preserved at −80°C until utilized for RNA extraction. Further comprehensive information can be found elsewhere (Tizioto et al., 2015). From the same population, 200 samples were selected for gene expression analysis by RNA-sequencing approach (RNA-Seq) because of financial limitations. This selected subsampling consisted of contrasting samples for phenotypes of interest.

### 2.3 Phenotypes and contrasting sample groups

The phenotypes were measured either during the feedlot phase or after the slaughter. Genomic estimated breeding values (GEBV), contrasting sample groups, and differentially expressed genes (DEGs) for each phenotype were previously identified (Cesar et al., 2015; Cesar et al., 2016; Tizioto et al., 2016; Silva-Vignato et al., 2017; Gonçalves et al., 2018) and used here as comparison with our genes of interest. The meat production phenotype under consideration is residual feed intake (RFI), the carcass phenotypes considered are backfat thickness (BFT) and ribeye area (REA) and the beef quality phenotypes considered are tenderness (TS), intramuscular fat (IMF), conjugated linoleic acid (CLA), oleic acid (OA), palmitic acid (PA), eicosapentaenoic acid (EPA) and docosahexaenoic acid (DHA). The distribution of samples across contrasting groups, based on the GEBV for each phenotype and including those with RNA-Seq data, was as follows: 20 for RFI, six for BFT and REA, 24 for TS, seven for IMF, and 30 for CLA, OA, PA, EPA, and DHA, being half for the high group and half for the low group for each phenotype. The groups are the same as previously published with the differential expression results. To allow comparisons, contrasting groups, necessary for the next analysis, comprised the same samples as the ones included in previous publications regarding the same phenotype. The significancy of GEBVs’ differences between contrasting groups was tested before further analysis. There is no complete overlap between contrasting groups of different traits, being four the maximum number of samples overlapping contrasting groups, being common for the high groups of DHA and EPA.

### 2.4 Expression data

Total RNA extraction from the 200 samples was described previously (Da Silva Diniz et al., 2016). Briefly, 100 mg of frozen tissue per sample were used for RNA isolation using TRIzol reagent (Life Technologies, Carlsbad, CA). The RNA integrity was assessed using a Bioanalyzer 2100 (Agilent, Santa Clara, CA, United States). For samples with an RNA integrity number (RIN) > 8, a total of 2 μg of RNA was utilized for library preparation following the TrueSeq RNA Sample Preparation Kit v2 guidelines (Illumina, San Diego, CA). Library integrity was validated using the Bioanalyzer 2100, while quantification was performed using quantitative PCR with the KAPA Library Quantification Kit (KAPA Biosystems, Foster City, CA, United States).

The library of the 200 samples were sequenced, wherein each batch consisted of three pools, with six samples per pool, occupying three clustered lanes on a sequencing flow cell. Clustering was accomplished utilizing the TruSeq PE Cluster Kit v3-cBot-HS Kit (Illumina, San Diego, CA, United States), and sequencing was performed on a HiSeq2500 ultrahigh-throughput sequencing system (Illumina, San Diego, CA, United States) employing the TruSeq SBS Kit v3-HS (200 cycles) at the Genomics Center at ESALQ, Piracicaba, São Paulo, Brazil.

The raw reads were analyzed as described before (Afonso et al., 2020). In summary, reads with a length greater than 65 bp and a Phred score greater than 24 were aligned and quantified considering the *Bos taurus* reference genome (ARS-UCD 1.2) using STAR software v.2.5.4 (Dobin et al., 2013). Genes not expressed in at least 20% of the samples were filtered from the expression dataset and normalization was performed with the VST function from DESeq2 software (Love et al., 2014). Batch effect combinations of sequencing flow cells and lanes were adjusted with the ARSyNseq function of NOISeq software v.2.16.0 (Tarazona et al., 2015).

(Cesar et al., 2015; Cesar et al., 2016; Tizioto et al., 2016; Silva-Vignato et al., 2017; Gonçalves et al., 2018).

### 2.5 Identification of discordantly regulated genes (DRGs)

As described in our previous work (Afonso et al., 2020), we employed an *in-silico* approach utilizing the TRIAGE method (Shim et al., 2020) with the RankProd R package (Hong et al., 2006) to identify discordantly regulated genes (DRGs) for each phenotype. The TRIAGE method identifies genes with potential epigenetic regulation in each sample, most probably being epigenetically regulated in a particular sample. These genes are the best ranked based on a discordant score, calculated by the multiplication of the specific gene repressive tendency score (given by the TRIAGE authors) and the gene expression value. The RankProd R package compares the ranks from contrasting sample groups to identify the ones differentially ranked between the groups. DRGs are candidate regulators for each phenotype that are potentially influenced by epigenetic mechanisms, inferred from the expression data.

The TRIAGE method incorporates a repressive tendency score assigned to each gene, which was initially estimated from human data (Shim et al., 2020). We considered the same repressive tendency score for *Bos taurus* based on orthologous genes (one-to-one) annotated using BiomaRt software (Ensembl genes 112) (Smedley et al., 2015). Among the 16,704 genes known to be orthologous one-one-one between *Bos taurus* and humans on Ensembl genes 112, 13,483 genes were expressed in our samples. From these, 635 genes contain repressive tendency scores for humans and were used to following analysis. The number of genes decreased a lot in this step because only genes with a known repression by epigenetic in humans have repressive tendency scores. The usage of only orthologous one-to-one between *Bos taurus* and humans is a limitation of the technique. But the small number of orthologous genes that contain repressive tendency scores compared to the real number of orthologous genes is not a limitation. It is an advantage, since only the genes that are affected by epigenetic repression in human tissues are considered, what decreases the chances of false positives.

We calculated the discordance score for each of the 635 selected genes, defined as the product of their logarithmic expression and its repressive tendency score, according to the TRIAGE methodology. The genes for each sample were then ranked based on their discordance scores across samples, from the biggest values to the smallest values, with higher-ranked genes deemed more susceptible to epigenetic regulation. Subsequently, to identify DRGs associated with each phenotype, we compared gene rankings between contrasting sample groups for each phenotype, using the RankProd R-package (Hong et al., 2006). This package utilizes a non-parametric method based on the estimated percentage of false predictions (pfp). We used a threshold of pfp >0.1 to consider the position of a specific gene in the rank for a contrasting sample group different from the position of the same gene in the rank for the other contrasting sample group. The less conservative threshold was chosen because the method is new. We investigated known bovine transcription factors (TFs) and transcription co-factors (TcoFs) among the DRGs using the Bovine TF database from AnimalTFdb v. 4.023 (Shen et al., 2023) and confirmed the annotation of these TFs and TcoFs in a Compendium of bovine TFs (de Souza et al., 2018) or in the UniProt database (Bateman et al., 2025). For the functional annotation, DRGs across all phenotypes were subjected together to analysis using STRING software (Szklarczyk et al., 2019). Given the extensive range of biological processes, REVIGO software (Supek et al., 2011) was used to summarize related Gene Ontology (GO) terms. Lastly, we cross-referenced the DRGs to previously reported DEGs for the same phenotypes (Cesar et al., 2015; Cesar et al., 2016; Tizioto et al., 2016; Silva-Vignato et al., 2017; Gonçalves et al., 2018).

### 2.6 Candidate SNPs associated with traits and epigenetic regulation

To investigate the association between single nucleotide polymorphisms (SNPs) located proximal to or within the DRGs and their respective phenotypes, based on literature about the range of histone modification effects (Barski et al., 2007), we scanned a window of 10 kb flanking each side of the transcription start site (TSS) for every DRG. These regions were assessed for their association with the GEBV of each phenotype utilizing PLINK software (Purcell et al., 2007). We used a subset of 104 samples for this analysis because these are the ones containing RNA-Seq and phenotype data for all traits in study. SNPs of interest were annotated using the VEP software v.110 (McLaren et al., 2010) and an analysis of variance (ANOVA) was conducted to compare GEBVs among genotype groups formed by SNPs associated with at least one phenotype.

To investigate potential epigenetic regulation within the genomic regions harbouring the trait-associated SNPs, we searched for peaks in ATAC-Seq and ChIP-Seq data available for two bovine muscle samples from the Functional Annotation of Animal Genomes (FAANG) project, accessed through the UCSC Genome Browser on Cow (April 2018, version ARS-UCD1.2/bos Tau9 (Nassar et al., 2023)). Furthermore, the cattleQTLdb database (Hu et al., 2013) was used to identify bovine traits associated with the SNPs of interest.

### 2.7 Regulatory impact of the DRG on phenotypes

Given that DRGs are expected to act as regulators of traits while also being subjected to epigenetic control, we searched for additional evidence of the regulatory impact of DRGs and DEGs on traits via the Regulatory impact factors algorithm (RIF) (Reverter et al., 2010). The RIF algorithm was designed to infer the regulatory impact of TFs (potential regulators) on the expression of selected gene targets (potential targets of the regulation), based on their expression values. Herein we used RIF to infer the regulatory impact of DRGs and DEGs on traits. We used the DRGs’ and DEGs’ expression values as the potential regulators and traits’ GEBVs as the potential targets of regulation. For each trait, we considered the DRGs and DEGs’ expression identified, contrasting sample groups and run the RIF algorithm separately. Table 1 contains the number of DRGs and DEGs used in each run for each trait. The DRGs and/or DEGs presenting RIF 1 or RIF 2 scores higher than |1.96| are genes with predicted regulatory impact over a specific trait, thus called a RIF gene for this trait. RIF1 score give high scores (positives or negatives) to candidate regulators that are most differentially co-expressed, highly abundant and with more expression differences between the contrasting groups. It searches for the candidate regulators that seem to have expression differences between the contrasting groups per trait. RIF2 score gives high scores (positives or negatives) to candidate regulators which expression can predict better the abundance of the genes in the target lists.

**TABLE 1:** Number of Differential Regulated Genes, SNPs and Differentially Expressed Genes identified or used in the analysis for each trait.

Type of trait	Trait	DRG^a^	SNPs^b^	DEG^c^
Production	RFI	15	618	58
Carcass	BFT	1	45	15
	REA	6	156	91
Quality	TS	7	125	11
	IMF	5	113	63
	CLA	24	796	707
	OA	32	1204	970
	PA	26	784	96
	EPA	22	568	4
	DHA	22	671	2

^a^ number of DRGs, identified.

^b^ number of SNPs, considered for the association test between SNPs, flanking the TSS, of DRGs, and the given trait.

^c^ number of DEGs, considered for the RIF, analysis per trait. Numbers of DEGs, are smaller than reported in the original publications due to annotation changes in the latest reference genome. RFI, residual feed intake; BFT, backfat thickness; REA, ribeye area; TS, tenderness; IMF, intramuscular fat; CLA, conjugated linoleic acid; OA = oleic acid; PA, palmitic acid; EPA, eicosapentaenoic acid; DHA, docosahexaenoic acid.

Additionally, we explored the relationships among the expression patterns of DRGs, previously published DEGs and the GEBV of each of the traits through correlation analysis performed with the Partial correlation and information theory algorithm (PCIT) (Reverter and Chan, 2008). Phenotypic data, DRGs, previously reported DEGs and RIF genes, as well as known TFs and TcoFs for *Bos taurus*, were utilized as attributes for constructing the networks. Networks were created separately for each trait.

## 3 Results

### 3.1 Discordantly regulated genes (DRGs)

The DRGs for each production, carcass and beef quality trait identified here are candidate regulators of these traits, potentially modulated by epigenetic mechanisms (Afonso et al., 2023), as identified through a combination of the TRIAGE method and a rank product analysis (Hong et al., 2006).

In total, we identified 51 DRGs across all traits. Table 1 shows the number of DRGs identified for each one of the production, carcass and beef quality traits. Among these, 11 were DRGs for six or more traits (*i.e., LBX1, SIM2, HOXC10, PAX7, COMP, BTNL9, CDH22, EN1, ZIC4, TBX15* and *TBX3*). *LBX1* was identified as a DRG for all traits except BFT. Supplementary Figure S1 illustrates the DRGs for each trait with percentage of false positives (pfp) < 0.01, and their expression differences between contrasting groups. Similarly, Supplementary Table S1 provides details of the DRGs identified for each trait and their significant pfp values.

**FIGURE 1:**
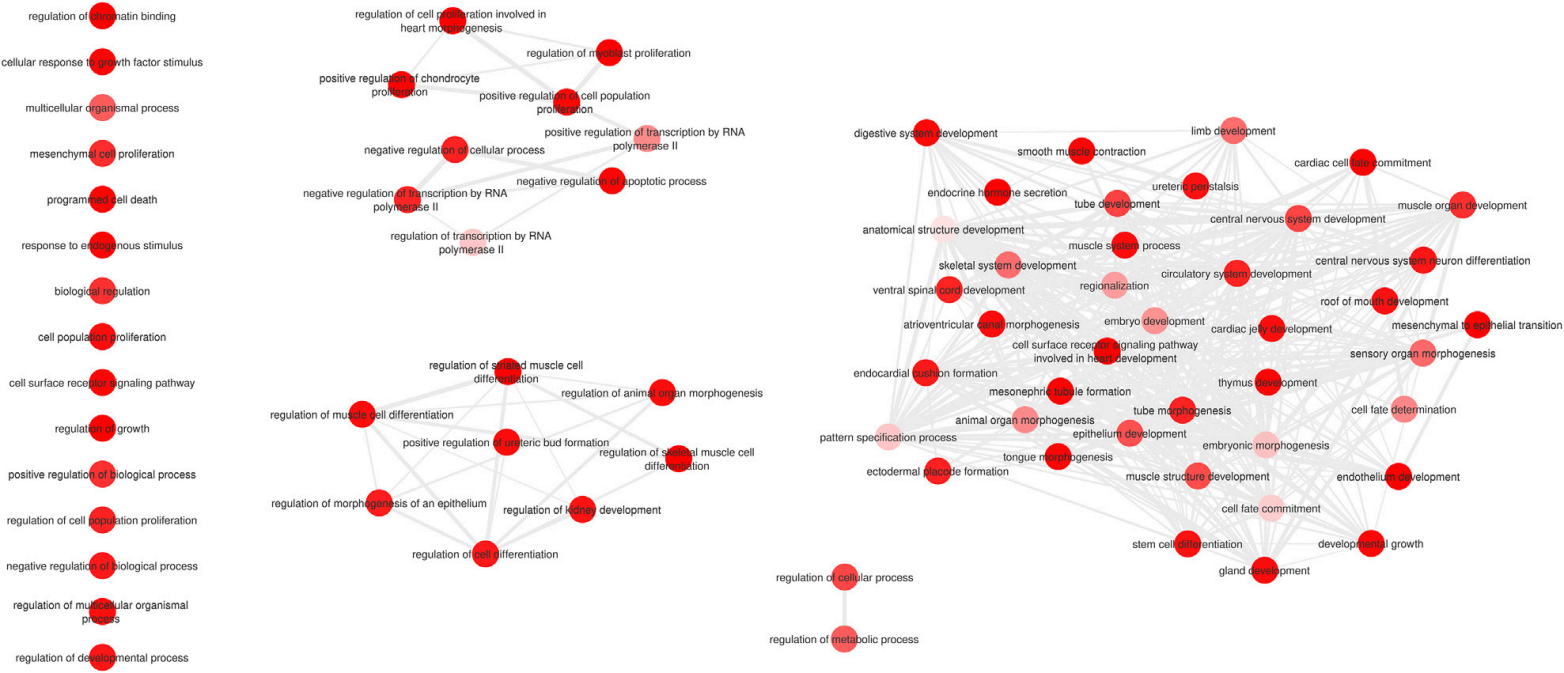
Clustered summary of Biological Processes enriched for all DRGs together. The summarizing and clustering were performed with the REVIGO software. The color intensity of the circles represent the FDR values for each GO term.

Among the 51 DRGs, 26 are known bovine TFs, 14 of which belong to the homeobox family. Only the *VGLL2* DRG is a known bovine TF co-factor (Supplementary Table S2). The collective DRGs across all phenotypes are implicated in 162 biological processes, that can be clustered and summarized in three cluster containing more than three processes and some processes alone (Figure 1). The biggest cluster is related to organ, system, and embryo development, while the three smallest ones are related to the regulation of transcription and cellular, molecular, and biological processes. Supplementary Table S3 presents the complete list of enriched biological processes for the DRGs (Supek et al., 2011). Eight DRGs were also DEGs in the same Nelore population, with six involved in at least one of the same traits and two with different traits. Table 2 shows the DRGs that are also DEGs and their respective traits.

**TABLE 2:** Genes that are both a DRG in our analysis and a DEG in previously published papers for the traits (Cesar et al., 2015; Cesar et al., 2016; Tizioto et al., 2016; Silva-Vignato et al., 2017; Gonçalves et al., 2018). Bold marks the genes being a DRG and a DEG or a DRG and a RIF for the same trait, and the trait in question is shown.

Gene	DRG	DEG	RIF	TF
*BARX1*	CLA	CLA		TF
*CDH22*	RFI, REA, CLA, AO, PA, EPA	CLA		
*MAFB*	AO, PA	CLA		TF
*PEBP4*	CLA, EPA	CLA		
*SIX2*	RFI, CLA, AO, EPA, DHA	CLA		TF
*GRM4*	EPA, DHA	IMF		
*HOXC10*	RFI, TS, IMF, CLA, AO, PA, EPA, DHA	RFI	IMF	TF
*CHRND*	RFI, AO, DHA	RFI, BF		

### 3.2 Candidate SNPs associated with traits and epigenetic regulation

Our hypothesis was that some SNPs proximal to the TSS of DRGs are associated with the traits and contain peaks of epigenetic marks. To identify these SNPs, we performed an association analysis between the SNPs flanking the TSS of each DRG and the variation in the GEBV for these traits, utilizing a dataset of 104 samples. These samples were chosen among the 200 samples with RNA-Seq data because they also had genotype and complete phenotype data. Table 1 shows the number of SNPs used in the association tests for each trait.

We identified one SNP associated with CLA and 35 SNPs associated with BFT (Table 3). No SNPs associated with the other eight phenotypes were identified. The SNP associated with CLA was located within an intron of the *PITX2* DRG, which is located 7,537 bp downstream of its TSS. *PITX2,* a DRG for the traits CLA, OA, PA, EPA and DHA is also a known TF. Using an ANOVA single factor analysis, we found that the GEBV for CLA was significantly different between genotype groups for the SNP associated with CLA (p-value = 3.56E-05). Table 4 shows the number of animals with each of the three genotypes for this SNP and the mean CLA GEBV per genotype. The allele substitution effect for this SNP was −0.0072, indicating that substituting a T allele with a C allele is associated with a decrease of 0.0072 in the GEBV population mean for CLA.

**TABLE 3:** SNPs around the DRGs associated with the traits in the present study (FDR_BH < 0.05). The tested SNPs were positioned in a window of 10 kbp for each side of the TSS of the DRGs.

Phenotype	SNP Id	Location	FDR_BH	Gene	Consequence	LD group^a^
CLA	rs110498194	6:14838839	0.005209	*PITX2*	intron_variant	
BFT	rs378651334	7:40077296	0.02457	-	intergenic_variant	A
BFT	-	7:40078493	0.03234	-	intergenic_variant	B
BFT	rs384819568	7:40079097	0.03234	-	intergenic_variant	
BFT	rs137771013	7:40079115	0.03234	-	intergenic_variant	
BFT	rs385960017	7:40079225	0.03234	-	intergenic_variant	
BFT	rs383582880	7:40079371	0.03234	-	intergenic_variant	
BFT	rs380982868	7:40079507	0.03234	-	intergenic_variant	
BFT	rs378164995	7:40079621	0.03234	-	intergenic_variant	
BFT	rs384405352	7:40079701	0.03234	-	intergenic_variant	
BFT	rs379971324	7:40079753	0.03234	-	intergenic_variant	
BFT	-	7:40079779	0.03234	-	intergenic_variant	
BFT	-	7:40079847	0.03234	-	intergenic_variant	
BFT	-	7:40079862	0.03234	-	intergenic_variant	
BFT	rs380713416	7:40080007	0.03234	-	intergenic_variant	
BFT	rs383673032	7:40080025	0.02457	-	intergenic_variant	
BFT	rs380831736	7:40080089	0.03234	-	intergenic_variant	
BFT	rs383200546	7:40080092	0.03234	-	intergenic_variant	
BFT	rs379943783	7:40080275	0.03234	-	intergenic_variant	
BFT	rs133293115	7:40080326	0.03234	-	intergenic_variant	
BFT	-	7:40080662	0.0333	-	intergenic_variant	
BFT	rs207501632	7:40080704	0.03234	-	intergenic_variant	
BFT	-	7:40081355	0.03234	-	intergenic_variant	
BFT	-	7:40083511	0.03234	*BTNL9*	upstream_gene_variant	
BFT	rs385820179	7:40084046	0.03234	*BTNL9*	upstream_gene_variant	
BFT	rs380244948	7:40087148	0.02457	*BTNL9*	intron_variant and upstrem_gene_variant	
BFT	rs381539702	7:40088197	0.02457	*BTNL9*	intron_variant and upstrem_gene_variant	
BFT	rs383247709	7:40088215	0.02457	*BTNL9*	intron_variant and upstrem_gene_variant	
BFT	rs207985633	7:40089169	0.02457	*BTNL9*	intron_variant and upstrem_gene_variant	
BFT	rs210915265	7:40089363	0.02457	*BTNL9*	intron_variant and upstrem_gene_variant	
BFT	-	7:40089715	0.03106	*BTNL9*	intron_variant and upstrem_gene_variant	C
BFT	-	7:40091755	0.02457	*BTNL9*	intron_variant and upstrem_gene_variant	D
BFT	-	7:40092439	0.03234	*BTNL9*	intron_variant	E
BFT	-	7:40093135	0.02457	*BTNL9*	intron_variant	
BFT	-	7:40093821	0.02457	*BTNL9*	intron_variant	
BFT	-	7:40095095	0.03234	*BTNL9*	missense_variant	

a linkage desequilibrium group containing the SNPs.

**TABLE 4:** Details about the four genotypes of the SNP associated with CLA GEBV.

Genotype	Number of animals	Average of CLA GEBV
Homozygous alternative (C/C)	68	−0.0018
Heterozygous (C/T or T/C)	30	0.0032
Homozygous reference (T/T)	6	0.0104

The SNPs associated with BFT were located within a 17,799 bp region encompassing variants around the *BTNL9* DRG, on chromosome six. The first SNP associated with BFT was positioned 9,331 bp upstream of the *BTNL9* gene, while the last SNP, a missense variant, is located 8,468 bp downstream of the *BTNL9* gene. This region comprises the proximal region, promoter, and part of the gene *BTNL9*, which was a DRG for BFT, RFI, OA, PA and EPA. Based on a linkage disequilibrium threshold of 0.8, this region can be divided into five groups (Table 3). No significant difference was detected between the genotype groups according to the ANOVA, considering one tag SNP per group.

**FIGURE 2:**
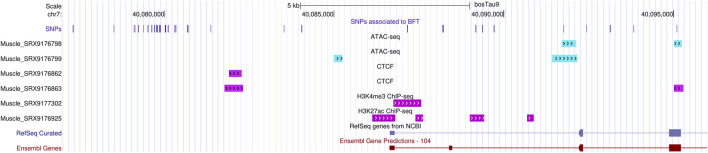
FAANG ATAC-Seq and ChIP-Seq for two muscle samples from cattle in the region encompassing the 35 SNPs associated with BFT. The information was gathered at UCSC Genome Browser on Cow. The first SNP was on chr7:40,077,296 and the last SNP was on chr7:40,095,095.

**FIGURE 3:**
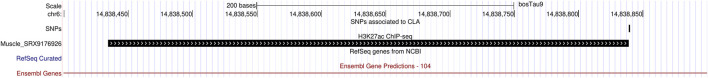
FAANG ATAC-Seq and ChIP-Seq for two muscle samples from cattle in the region encompassing the SNP associated with CLA. The information was gathered at the UCSC Genome Browser on Cow. The SNP is in chr6:14,838,839, an intron of the *PITX2* gene (a TF).

To investigate signs of epigenetic regulation within regions containing SNPs associated with traits, we examined ATAC-Seq and ChIP-Seq peaks for various marks, obtained from published data from the FAANG project. The region encompassing the 35 SNPs associated to BFT exhibited peaks for ATAC-Seq and ChIP-Seq for CTCF, H3K4me3 and H3K27acand marks, suggesting epigenetic regulation at these loci (Figure 2). The SNP associated with CLA is exactly in the end of a broad peak for H3K27ac (Figure 3), also suggesting epigenetic regulation there.

Considering QTLs associated with traits related to fatty acid deposition in the cattleQTLdb (Hu et al., 2013), the SNP associated with CLA (rs110498194) was found to be linked to body and carcass weight, fat thickness and fatty acid content in tissues and in milk for several cattle breeds (Supplementary Table S4). Similarly, the 35 SNPs associated with BFT were associated with marbling score, fat thickness, and body weight in the Angus and Hanwoo breeds (Supplementary Table S5).

### 3.3 Regulatory impact of the DRG on phenotypes

As DRGs are proposed regulators of traits that are also under epigenetic control, we investigated their regulatory impact, along with DEGs described in previous studies and already associated with the traits, utilizing the RIF algorithm. Table 1 shows the number of DRGs and DEGs tested per trait. We identified nine RIF genes for RFI, two for BFT, nine for REA, two for TS, five for IMF, 73 for CLA, 71 for OA, 11 for PA, one for EPA and three for DHA. Four genes were RIF genes for multiple traits: *IFNLR1* for CLA and PA, *COL2A1* for REA and DHA and *SIM2* and 3*VGLL2* for OA and PA. Among the RIF genes, 12 were DRGs, one was both a DRG and a DEG and 169 were DEGs. Supplementary Table S6 provides a comprehensive list of all the RIF genes, indicating DRGs and DEGs, as well as the traits on which they were identified. Among all the RIF genes, 25 were TFs. Among them, *HOXC10* had the most attributes linked to the studied traits, being a DRG for RFI, TS, IMF, CLA, OA, PA, EPA and DHA, a DEG for RFI and a TF.

Additionally, we explored the relationships among the expression of DRGs, previously published DEGs in this same population and the variation in the GEBV of each of the traits via correlation analysis conducted with the PCIT algorithm. Supplementary Figure S2 presents the networks constructed based on these correlations, with each network focusing on a specific trait. Phenotypes, DRGs, previously published DEGs, RIF genes, and known TFs and TcoFs for *Bos taurus* are marked within the networks. We can see in all the networks that there is an intricate relationship among DEGs and DRGs. Interestingly, the EPA and DHA networks show that a bunch of DRGs are correlated with just four DEGs for EPA and just two DEGs for DHA. The gene *PITX2*, containing the only SNP associated to CLA, does not have its expression correlated directly to CLA GEBV, but it is second neighbour of the CLA GEBV in the network, being correlated to two DRGs that are also TFs (*BARX1* and *SIX2*) and two DEGs (*NOD1* and *ADAMTS3*). As for the gene *BTNL9*, containing most of the group of SNPs associated with BFT, its expression is also second neighbour to the BFT GEBV, being correlated directly to the DEG *DRP2*.

## 4 Discussion

### 4.1 Putative regulators of traits also subjected to epigenetic regulation

To elucidate the complex regulation of production, carcass and beef quality traits, understanding their epigenetic regulation is crucial. However, experiments targeting epigenetic markers can be complex and expensive, therefore alternative approaches using more cost-effective data are needed. In this regard, the use of the TRIAGE method and the RankProd R package, as previously published (Afonso et al., 2020), offers an opportunity to predict regulatory genes affecting traits while also being regulated by epigenetic mechanisms (DRGs). This prediction, in the TRIAGE method, is based on the inverse relationship between the presence of the histone modification H3K27me3 and gene expression, coupled with a comparison of repression gene ranks between contrasting sample groups. The TRIAGE method primarily relies on the presence of H3K27me3 histone modifications in various human tissues. However, the authors validated its capacity to identify epigenetic regulation in other species, identify genes being affected by epigenetic variables and potentially mediated by different mechanisms beyond H3K27me3 alone (Shim et al., 2020). To use TRIAGE with bovine expression data, we use ortholog genes between bovines and humans, as the authors suggest. Here, DRGs provide insights into the regulation of complex production, carcass and beef quality traits.

Considering all the traits studied here, the *LBX1* gene is one of the main regulatory candidates for production, carcass and beef quality traits since it was a DRG for all traits, except for BFT. This gene encodes a TF, which is a candidate regulator of fatty acid composition-related traits (Valdés-Hernández et al., 2024) and associated with feed conversion ratio in pigs. A SNP in the *LBX1* gene has been associated with idiopathic scoliosis in humans, a disease that causes muscle atrophy (Shahidi et al., 2021). Based on that, we can speculate that this TF would regulate processes underlying muscle fibre size, function and tenderness. Among DRGs associated with at least six traits, *HOXC10* was associated with eight traits and *CDH22* was associated with six traits, respectively, showing evidence of their involvement with these traits. The known functions of all DRGs, based on the functional enrichment analysis, are linked to embryo development and the regulation of biological processes, both of which are acknowledged as being subject to epigenetic regulation.

### 4.2 Supporting evidence of the regulatory status, epigenetic regulation of DRGs and relationship with the traits

We searched for additional evidence that the DRGs are trait regulators. Apart from our results with the TRIAGE method coupled with the RankProd R package, we have supporting evidence suggesting that DRGs serve as candidate regulators of the studied traits. First, 50.9% of the DRGs were TFs, whereas only 8.45% of all the genes in the genome used for DRGs identification were TFs. The overrepresentation of TF among the DRG compared to what would be expected at random strongly supports the regulatory role DRGs. Second, SNPs located within or around DRGs were associated with the phenotypes, with the allele substitution of one SNP associated with CLA being associated with a decrease of 0.0072 in the GEBV of this phenotype. Accordingly, genetic variants within epigenetically regulated regions of the genome, such as those influenced by histone modifications, may be correlated with certain traits (Kang et al., 2021; Kang et al., 2021). *PITX2* is a TF found as DRG, that contains the SNP associated with CLA content in muscle. *PITX2* is involved in the fine-tuning mechanism of skeletal-muscle development, differentiation and cell fate in adult muscle (Hernandez-Torres et al., 2017), which are functions frequently affected by epigenetics and with regulatory significance. Based on our search in the cattleQTLdb for the regions of or around the SNPs associated to our traits, we saw that SNPs near the one associated with CLA were QTL for body weight, fat thickness in several tissues, and also QTLs for fatty acid content and milk fat in several bovine breeds (Nassar et al., 2023). Similarly, SNPs around the *BTNL9* gene, which were associated here with BFT, were reported as QTLs for marbling score, fat thickness and body weight in the Angus and Hanwoo breeds (Hu et al., 2013). A human variant in the *BTNL9* gene is associated with both lower high-density lipoprotein cholesterol and greater triglycerides (Carlson et al., 2023), providing additional evidence of the link between this gene and fat deposition in our study. These SNPs in regions potentially affected by epigenetic mechanisms could serve as points of linkage between these mechanisms and the traits, although further studies are warranted to validate this hypothesis.

The third evidence is derived from the RIF analysis, which predicted that out of the 51 DRGs identified across all traits, 13 were identified as RIF genes, suggesting their regulatory impact on at least one trait. *HOXC10*, a DRG for eight traits, a DEG for RFI (Tizioto et al., 2016) and a TF, is a RIF gene with more attributes linking it to the traits in study. Together with *LBX1,* these genes were the two main candidate regulators of the traits studied here presenting epigenetic regulation. *HOXC10*, a homeobox gene linked to morphogenesis in multicellular organisms, has been associated with mineral content in Nelore muscle (Afonso et al., 2020) and was identified as a QTL region for chest depth in the Limousin breed (Doyle et al., 2020), underscoring its relevance to muscle and production, carcass and beef quality traits.

For the epigenetic regulation of the DRGs, the regions containing the 35 SNPs around DRGs associated with BFT were regions of open chromatin in bovine muscle samples from the FAANG project, indicating susceptibility to regulation. These regions exhibit ChIP-Seq peaks of various histone modifications in the same bovine muscle samples. The SNP associated with CLA, in an intron of the gene *PITX2* which is a known TF, is at the far end of a broad peak for H3K27ac. H3k27ac broad peaks in introns suggest enhancer activity, even more in TFs (Zhou et al., 2022). Future experiments involving for instance ChIP-Seq and ATAC-Seq on samples from contrasting groups are necessary to explore potential differences in epigenetic regulation affecting these regions between groups.

Finally, the network analysis revealed significant positive and negative correlations among DRGs, DEGs, and traits. These networks, one for each trait, showed intricate nets with DEGs and/or DRGs directly linked to traits and among themselves, including numerous TFs and TcoFs. The connections between RIF genes or DRGs and DEGs represent candidate regulatory events that warrant further exploration in future studies.

While our results unveiled the potential epigenetic blueprint of beef quality and production traits, it is important to acknowledge that TRIAGE includes only protein-coding putative regulators of the phenotypes undergoing regulation by epigenetic mechanisms, rather than all potential regulators; and relies on human data, restricting our ability to test genes to those exhibiting orthology between humans and cows.

### 4.3 Limitations of the study

This study constitutes an *in silico* and exploratory analysis aimed at initiating the investigation into the epigenetic regulation associated with various phenotypes in Nelore cattle. Our methodology, however, presents three limitations: (1) TRIAGE primarily focuses on genes highly involved in driving cell differentiation and lineage diversification, potentially introducing bias into the results. Nevertheless, this bias also serves as additional evidence of regulation. (2) TRIAGE exclusively examines protein-coding genes, implying that our findings only include protein-coding putative regulators of the phenotypes undergoing regulation by epigenetic mechanisms, rather than all potential regulators. (3) Since TRIAGE relies on human data, our ability to test genes is restricted to those exhibiting one-to-one orthology between humans and cows. Although wet laboratory validation, such as ChIP-Seq is needed to confirm these findings, our results will help to advance our understanding on the intricate epigenetic regulation linked to production and beef quality traits, improving animal production in the future.

## 5 Conclusion

We identified candidate genes potentially regulating beef quality and production traits subjected to epigenetic regulation (DRGs). We provided supporting evidence regarding the regulatory status of these DRGs, and their association with the studied traits. Additionally, we identified candidate SNPs potentially linking epigenetic mechanisms and the genome regulation, as well as putative regulatory events characterized by significant correlations among RIF genes, DEGs and DRGs. Among the identified DRGs, the TF *LBX1* and the gene *HOXC10* have emerged as candidates supporting their role as regulators of production, carcass and beef quality traits, while also being subjected to epigenetic regulation.

## Data Availability

Publicly available datasets were analyzed in this study. This data can be found here: The expression datasets supporting the results of this study are from an RNA-Seq experiment and are available in the ENA repository (EMBL-EBI), under study accession number PRJEB15314.

## References

[B1] Afonso J. Fortes M. R. S. Reverter A. Diniz W. J. da S. Cesar A. S. M. Lima A. O. de (2020). Genetic regulators of mineral amount in Nelore cattle muscle predicted by a new co-expression and regulatory impact factor approach. Sci. Rep. 10, 8436–16. 10.1038/s41598-020-65454-7 32439843 PMC7242321

[B2] Afonso J. Shim W. J. Boden M. Salinas Fortes M. R. da Silva Diniz W. J. de Lima A. O. (2023). Repressive epigenetic mechanisms, such as the H3K27me3 histone modification, were predicted to affect muscle gene expression and its mineral content in Nelore cattle. Biochem. Biophys. Rep. 33, 101420. 10.1016/j.bbrep.2023.101420 36654922 PMC9841166

[B3] Barski A. Cuddapah S. Cui K. Roh T. Y. Schones D. E. Wang Z. (2007). High-resolution profiling of histone methylations in the human genome. Cell 129, 823–837. 10.1016/j.cell.2007.05.009 17512414

[B4] Bateman A. Martin M.-J. Orchard S. Magrane M. Adesina A. Ahmad S. (2025). UniProt: the universal protein knowledgebase in 2025. Nucleic Acids Res. 53, D609–D617. 10.1093/nar/gkae1010 39552041 PMC11701636

[B5] Carlson J. C. Krishnan M. Rosenthal S. L. Russell E. M. Zhang J. Z. Hawley N. L. (2023). A stop-gain variant in BTNL9 is associated with atherogenic lipid profiles. Hum. Genet. Genomics Adv. 4, 100155. 10.1016/j.xhgg.2022.100155 PMC9630829 36340932

[B6] Cesar A. S. M. Regitano L. C. A. Koltes J. E. Fritz-Waters E. R. Lanna D. P. D. Gasparin G. (2015). Putative regulatory factors associated with intramuscular fat content. PLoS One 10, e0128350. 10.1371/journal.pone.0128350 26042666 PMC4456163

[B7] Cesar A. S. M. Regitano L. C. A. Poleti M. D. Andrade S. C. S. Tizioto P. C. Oliveira P. S. N. (2016). Differences in the skeletal muscle transcriptome profile associated with extreme values of fatty acids content. BMC Genomics 17, 961–16. 10.1186/s12864-016-3306-x 27875996 PMC5120530

[B8] Da Silva Diniz W. J. Coutinho L. L. Tizioto P. C. Cesar A. S. M. Gromboni C. F. Nogueira A. R. A. (2016). Iron content affects lipogenic gene expression in the muscle of Nelore beef cattle. PLoS One 11, e0161160–19. 10.1371/journal.pone.0161160 27532424 PMC4988672

[B9] de Souza M. M. Zerlotini A. Geistlinger L. Tizioto P. C. Taylor J. F. Rocha M. I. P. (2018). A comprehensive manually-curated compendium of bovine transcription factors. Sci. Rep. 8, 13747–12. 10.1038/s41598-018-32146-2 30213987 PMC6137171

[B10] Dobin A. Davis C. A. Schlesinger F. Drenkow J. Zaleski C. Jha S. (2013). STAR: ultrafast universal RNA-seq aligner. Bioinformatics 29, 15–21. 10.1093/bioinformatics/bts635 23104886 PMC3530905

[B11] Doyle J. L. Berry D. P. Veerkamp R. F. Carthy T. R. Walsh S. W. Evans R. D. (2020). Genomic regions associated with skeletal type traits in beef and dairy cattle are common to regions associated with carcass traits, feed intake and calving difficulty. Front. Genet. 11, 20. 10.3389/fgene.2020.00020 32117439 PMC7010604

[B12] Gonçalves T. M. De Almeida Regitano L. C. Koltes J. E. Cesar A. S. M. Da Silva Andrade S. C. Mourão G. B. (2018). Gene co-expression analysis indicates potential pathways and regulators of beef tenderness in Nellore cattle. Front. Genet. 9, 441–18. 10.3389/fgene.2018.00441 30344530 PMC6182065

[B13] Hernandez-Torres F. Rodríguez-Outeiriño L. Franco D. Aranega A. E. (2017). Pitx2 in embryonic and adult myogenesis. Front. Cell Dev. Biol. 5, 46. 10.3389/fcell.2017.00046 28507987 PMC5410577

[B14] Hong F. Breitling R. McEntee C. W. Wittner B. S. Nemhauser J. L. Chory J. (2006). RankProd: a bioconductor package for detecting differentially expressed genes in meta-analysis. Bioinformatics 22, 2825–2827. 10.1093/bioinformatics/btl476 16982708

[B15] Hu Z. L. Park C. A. Wu X. L. Reecy J. M. (2013). Animal QTLdb: an improved database tool for livestock animal QTL/association data dissemination in the post-genome era. Nucleic Acids Res. 41, 871–879. 10.1093/nar/gks1150 PMC3531174 23180796

[B16] Jaenisch R. Bird A. (2003). Epigenetic regulation of gene expression: how the genome integrates intrinsic and environmental signals. Nat. Genet. 33, 245–254. 10.1038/ng1089 12610534

[B17] Kang H. G. Lee Y. H. Lee S. Y. Choi J. E. Do S. K. Hong M. J. (2021). Genetic variants in histone modification regions are associated with the prognosis of lung adenocarcinoma. Sci. Rep. 11, 21520–10. 10.1038/s41598-021-00909-z 34728688 PMC8563968

[B18] Liotti A. Ferrara A. L. Loffredo S. Galdiero M. R. Varricchi G. Di Rella F. (2022). Epigenetics: an opportunity to shape innate and adaptive immune responses. Immunology 167, 451–470. 10.1111/imm.13571 36043705

[B19] Love M. I. Huber W. Anders S. (2014). Moderated estimation of fold change and dispersion for RNA-seq data with DESeq2. Genome Biol. 15, 550. 10.1186/s13059-014-0550-8 25516281 PMC4302049

[B20] McLaren W. Pritchard B. Rios D. Chen Y. Flicek P. Cunningham F. (2010). Deriving the consequences of genomic variants with the Ensembl API and SNP effect predictor. Bioinformatics 26, 2069–2070. 10.1093/bioinformatics/btq330 20562413 PMC2916720

[B21] Nassar L. R. Barber G. P. Benet-Pagès A. Casper J. Clawson H. Diekhans M. (2023). The UCSC Genome Browser database: 2023 update. Nucleic Acids Res. 51, D1188–D1195. 10.1093/nar/gkac1072 36420891 PMC9825520

[B22] Perrier A. Didelot A. Laurent-Puig P. Blons H. Garinet S. (2020). Epigenetic mechanisms of resistance to immune checkpoint inhibitors. Biomolecules 10, 1061–30. 10.3390/biom10071061 32708698 PMC7407667

[B23] Prowse-Wilkins C. P. Lopdell T. J. Xiang R. Vander Jagt C. J. Littlejohn M. D. Chamberlain A. J. (2022). Genetic variation in histone modifications and gene expression identifies regulatory variants in the mammary gland of cattle. BMC Genomics 23, 815. 10.1186/s12864-022-09002-9 36482302 PMC9733386

[B24] Purcell S. Neale B. Todd-Brown K. Thomas L. Ferreira M. A. R. Bender D. (2007). PLINK: a tool set for whole-genome association and population-based linkage analyses. Am. J. Hum. Genet. 81, 559–575. 10.1086/519795 17701901 PMC1950838

[B25] Reverter A. Chan E. K. F. (2008). Combining partial correlation and an information theory approach to the reversed engineering of gene co-expression networks. Bioinformatics 24, 2491–2497. 10.1093/bioinformatics/btn482 18784117

[B26] Reverter A. Hudson N. J. Nagaraj S. H. Pérez-Enciso M. Dalrymple B. P. (2010). Regulatory impact factors: unraveling the transcriptional regulation of complex traits from expression data. Bioinformatics 26, 896–904. 10.1093/bioinformatics/btq051 20144946

[B27] Shahidi B. Yoo A. Farnsworth C. Newton P. O. Ward S. R. (2021). Paraspinal muscle morphology and composition in adolescent idiopathic scoliosis: a histological analysis. JOR Spine 4, e1169. 10.1002/jsp2.1169 34611591 PMC8479518

[B28] Shen W. K. Chen S. Y. Gan Z. Q. Zhang Y. Z. Yue T. Chen M. M. (2023). AnimalTFDB 4.0: a comprehensive animal transcription factor database updated with variation and expression annotations. Nucleic Acids Res. 51, D39–D45. 10.1093/nar/gkac907 36268869 PMC9825474

[B29] Shim W. Sinniah E. Xu J. Vitrinel B. Alexanian M. Andreoletti G. (2020). Conserved epigenetic regulatory logic infers genes governing cell identity. Genes Gov. Cell Identity 11, 625–639.e13. 10.1016/j.cels.2020.11.001 PMC7781436 33278344

[B30] Silva-Vignato B. Coutinho L. L. Cesar A. S. M. Poleti M. D. Regitano L. C. A. Balieiro J. C. C. (2017). Comparative muscle transcriptome associated with carcass traits of Nellore cattle. BMC Genomics 18, 506–513. 10.1186/s12864-017-3897-x 28673252 PMC5496360

[B31] Smedley D. Haider S. Durinck S. Pandini L. Provero P. Allen J. (2015). The BioMart community portal: an innovative alternative to large, centralized data repositories. Nucleic Acids Res. 43, W589–W598. 10.1093/nar/gkv350 25897122 PMC4489294

[B32] Supek F. Bošnjak M. Škunca N. Šmuc T. (2011). Revigo summarizes and visualizes long lists of gene ontology terms. PLoS One 6, e21800. 10.1371/journal.pone.0021800 21789182 PMC3138752

[B33] Szklarczyk D. Gable A. L. Lyon D. Junge A. Wyder S. Huerta-Cepas J. (2019). STRING v11: protein-protein association networks with increased coverage, supporting functional discovery in genome-wide experimental datasets. Nucleic Acids Res. 47, D607–D613. 10.1093/nar/gky1131 30476243 PMC6323986

[B34] Tarazona S. Furió-Tarí P. Turrà D. Di Pietro A. Nueda M. J. Ferrer A. (2015). Data quality aware analysis of differential expression in RNA-seq with NOISeq R/Bioc package. Nucleic Acids Res. 43, e140. 10.1093/nar/gkv711 26184878 PMC4666377

[B35] Tizioto P. C. Coutinho L. L. Oliveira P. S. N. Cesar A. S. M. Diniz W. J. S. Lima A. O. (2016). Gene expression differences in Longissimus muscle of Nelore steers genetically divergent for residual feed intake. Sci. Rep. 6, 39493–12. 10.1038/srep39493 28004777 PMC5177880

[B36] Tizioto P. C. Gasparin G. Souza M. M. Mudadu M. A. Coutinho L. L. Mourão G. B. (2013). Identification of KCNJ11 as a functional candidate gene for bovine meat tenderness. Physiol. Genomics 45, 1215–1221. 10.1152/physiolgenomics.00137.2012 24151244

[B37] Tizioto P. C. Taylor J. F. Decker J. E. Gromboni C. F. Mudadu M. A. Schnabel R. D. (2015). Detection of quantitative trait loci for mineral content of Nelore longissimus dorsi muscle. Genet. Sel. Evol. 47, 15–19. 10.1186/s12711-014-0083-3 25880074 PMC4355494

[B38] Tollefsbol T. O. (2023). Handbook of epigenetics: the new molecular and medical genetics. Academic Press.

[B39] Valdés-Hernández J. Folch J. M. Crespo-Piazuelo D. Passols M. Sebastià C. Criado-Mesas L. (2024). Identification of candidate regulatory genes for intramuscular fatty acid composition in pigs by transcriptome analysis. Genet. Sel. Evol. 56, 12. 10.1186/s12711-024-00882-x 38347496 PMC10860264

[B40] Wei D. Li A. Zhao C. Wang H. Mei C. Khan R. (2018). Transcriptional regulation by CpG sites methylation in the core promoter region of the bovine SIX1 gene: roles of histone H4 and E2F2. Int. J. Mol. Sci. 19, 213. 10.3390/ijms19010213 29337851 PMC5796162

[B41] Yang Y. Zhou R. Mu Y. Hou X. Tang Z. Li K. (2016). Genome-wide analysis of DNA methylation in obese, lean, and miniature pig breeds. Sci. Rep. 6, 30160. 10.1038/srep30160 27444743 PMC4957084

[B42] Zhou Z. Jiang T. Zhu Y. Ling Z. Yang B. Huang L. (2022). A comparative investigation on H3K27ac enhancer activities in the brain and liver tissues between wild boars and domesticated pigs. Evol. Appl. 15, 1281–1290. 10.1111/eva.13461 36051459 PMC9423090

