# Supplementary material for: Putative epigenetic regulation mechanisms related to production, carcass and beef quality traits in Nelore cattle
Source: Front Genet. 2025 Jun 27;16:1593444. doi: 10.3389/fgene.2025.1593444 (PMC12245772; doi:10.3389/fgene.2025.1593444)
Supplement: Supplementary file 1 [file Supplementaryfile1.docx]

**Supplementary Figures**

**Supplementary Figure 1. Heatmaps representing the DRGs for each trait (pfp < 0.01).**

**
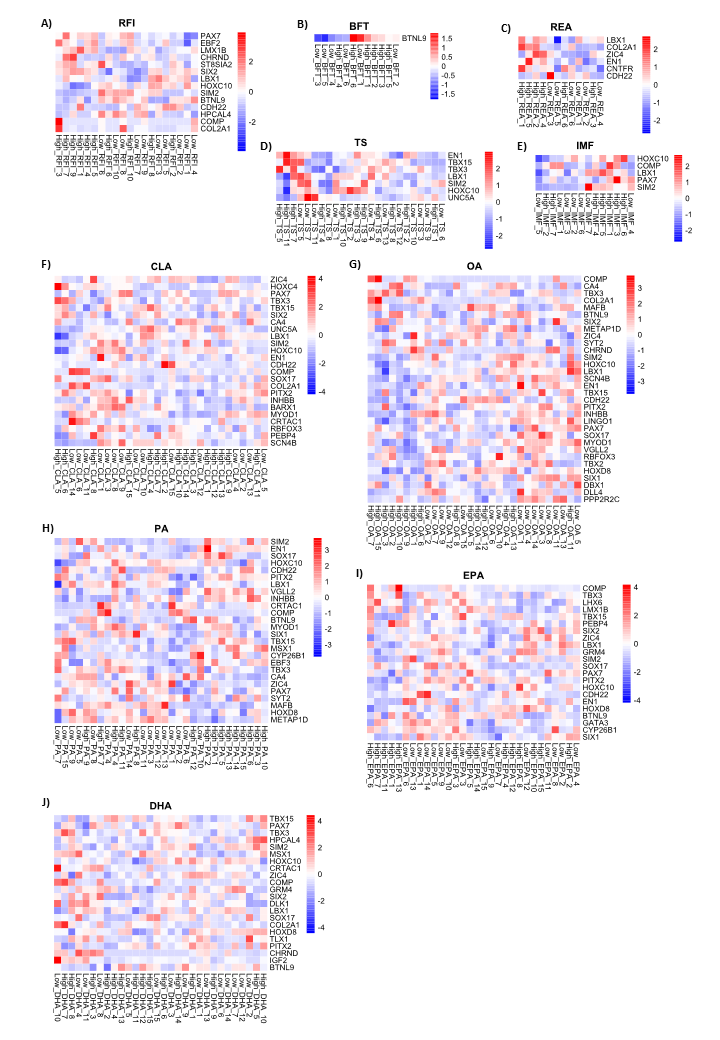
**

**Supplementary Figure 2. Networks, per phenotype, showing the significant correlations among DRGs, previously published DEGs and the GEBV of the phenotypes. A) Captions for all networks. B) RFI. C) BFT. D) REA. E) TS. F) IMF. G) CLA. H) OA. I) PA. J) EPA. K) DHA.**

1. **Captions for all networks**

**
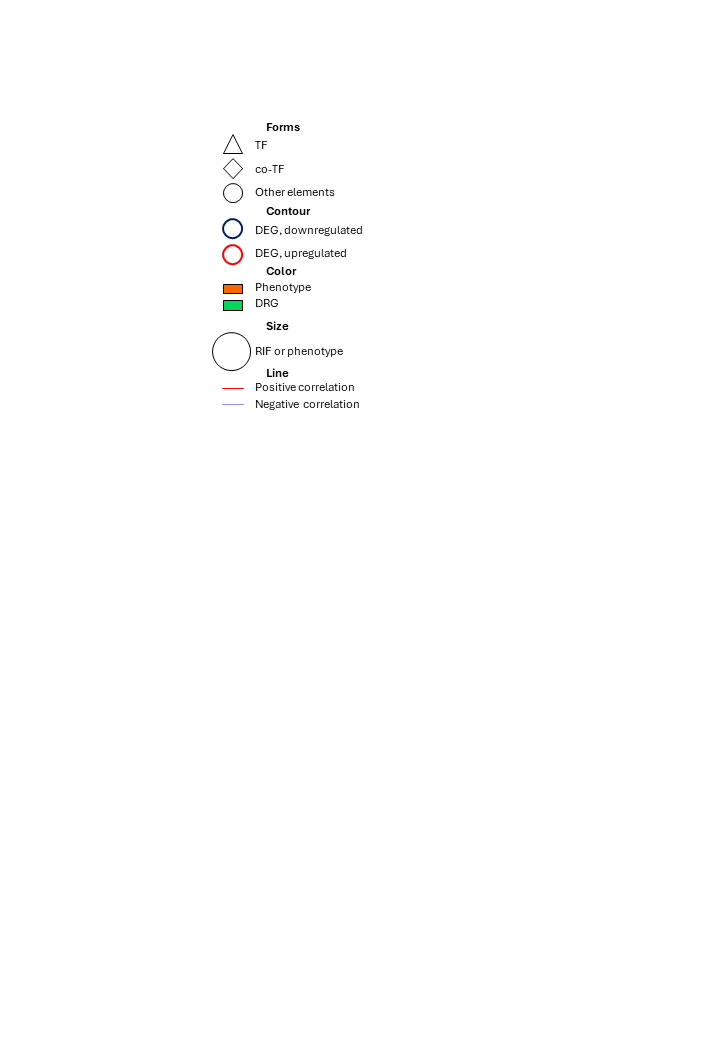
**

**B) RFI
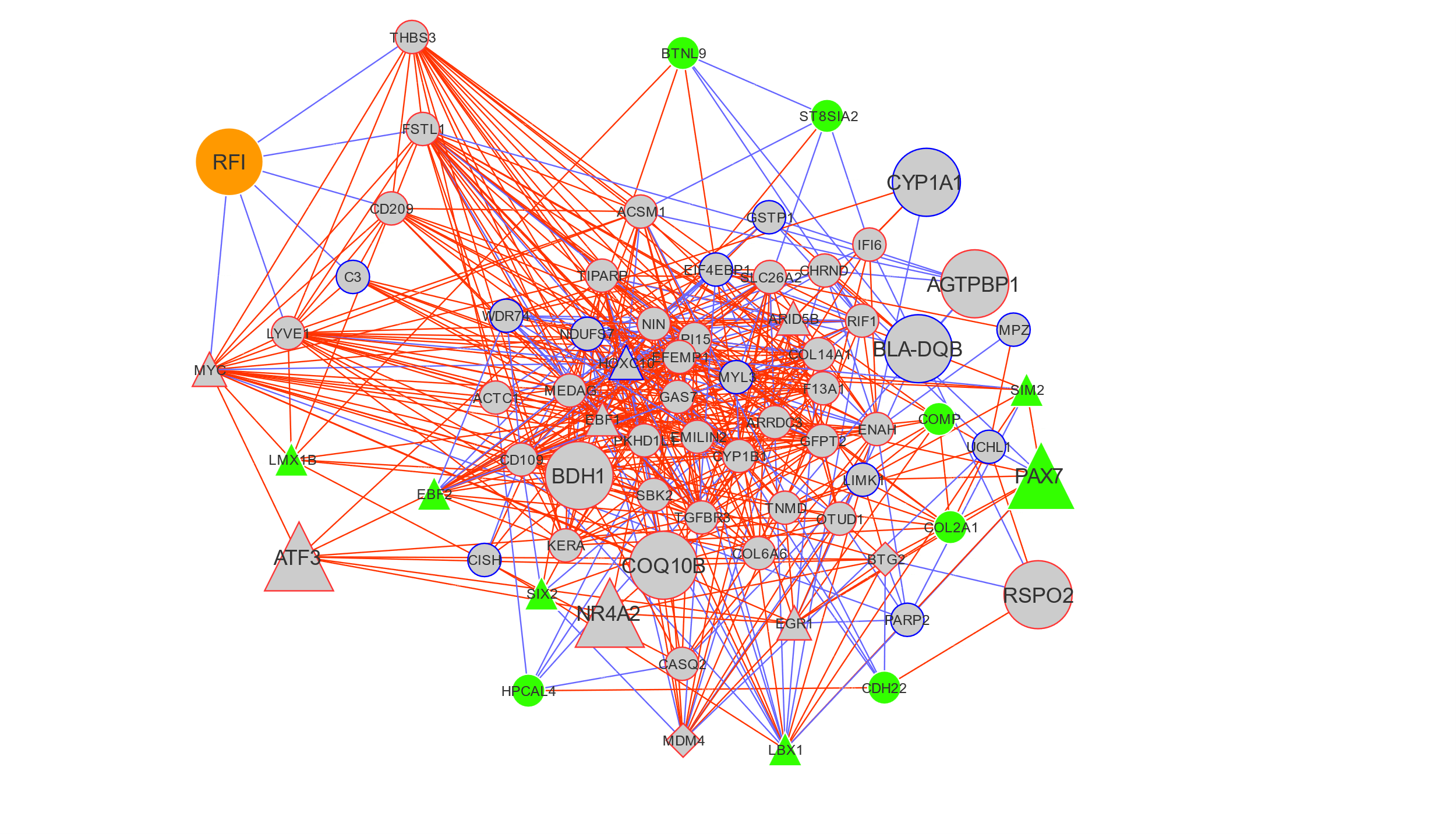
**

**C) BFT**

**
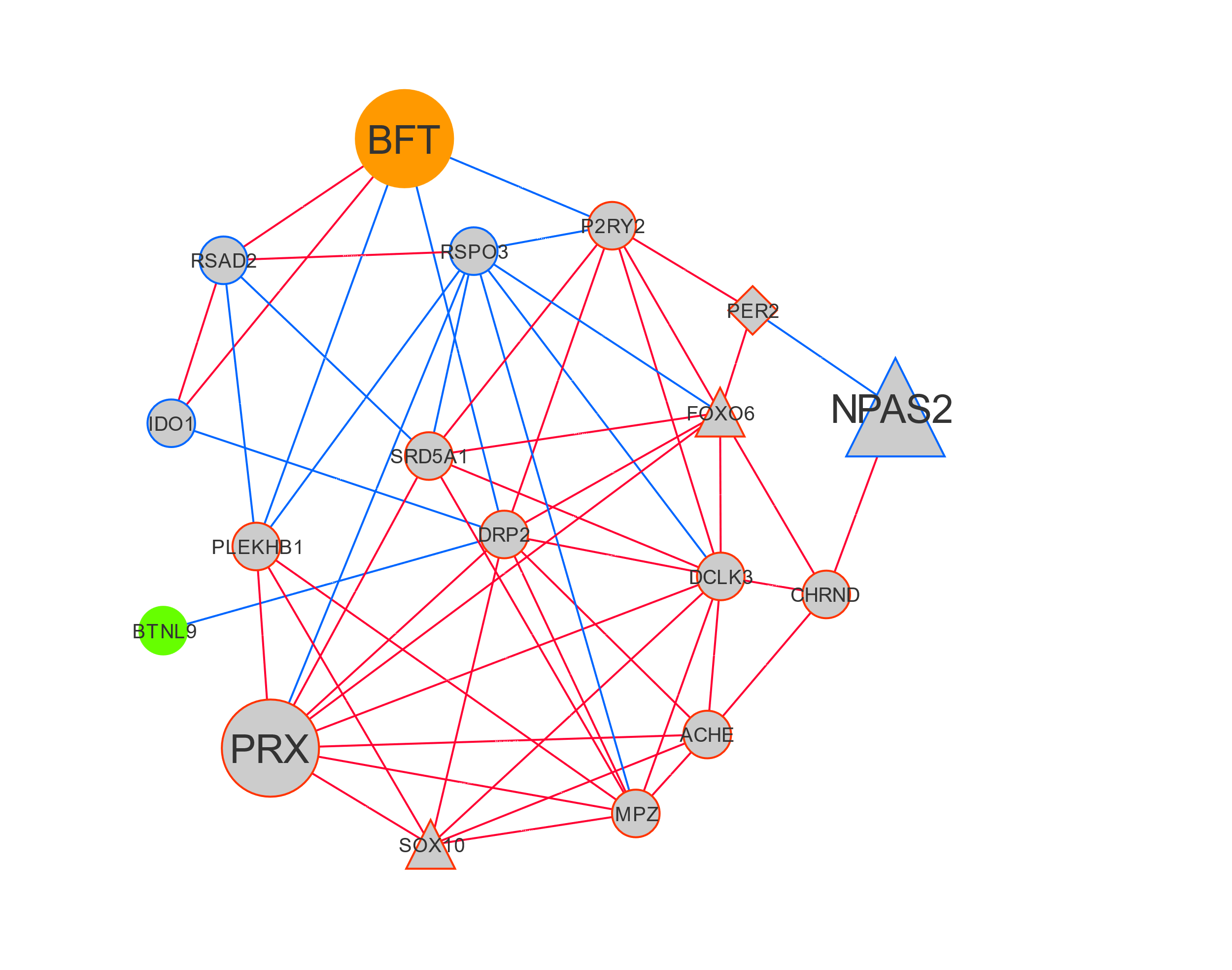
**

**D) REA**

**
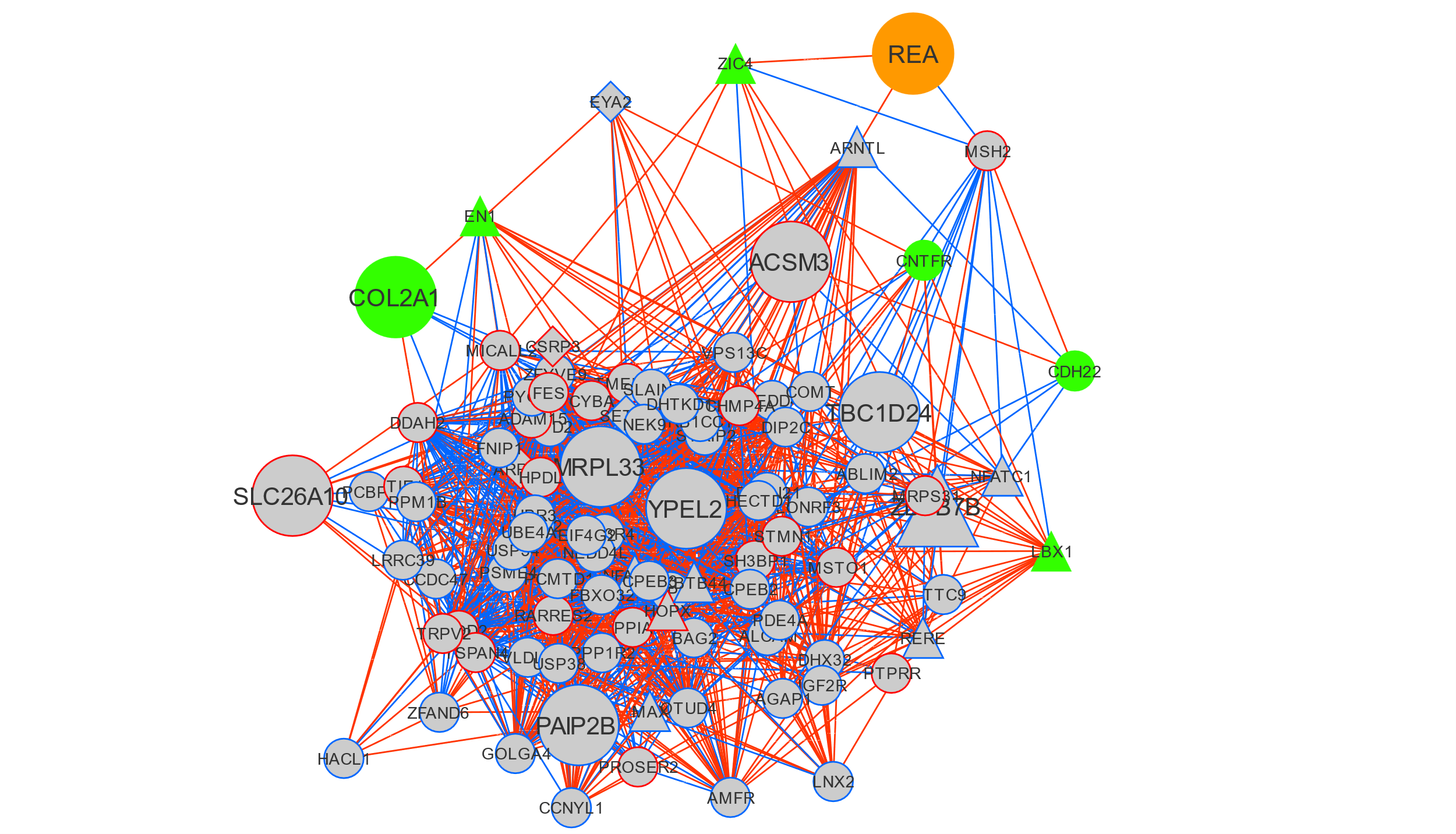
**

**E) TS**

**
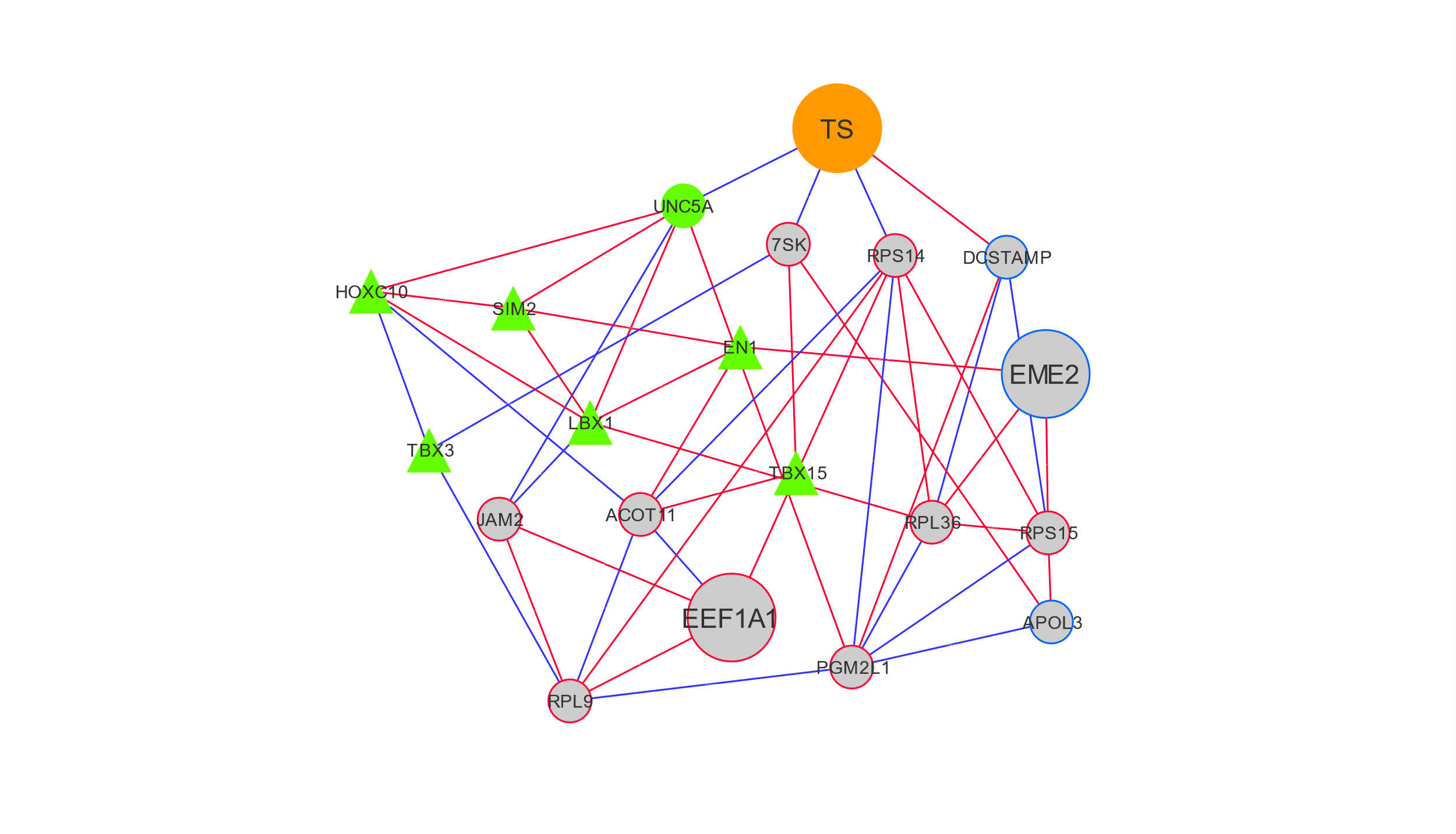
**

**F) IMF**

**
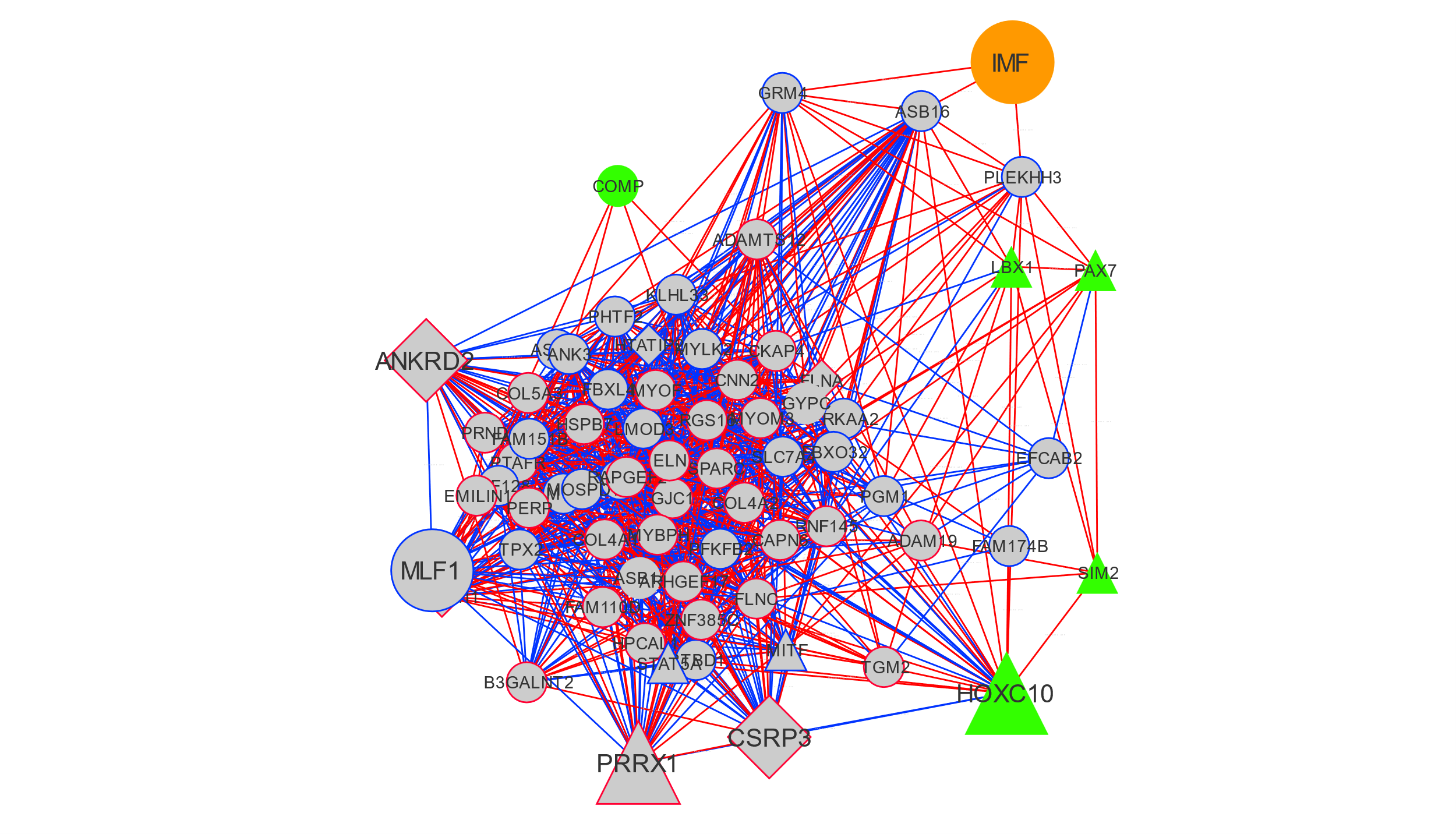
**

**G) CLA**

**
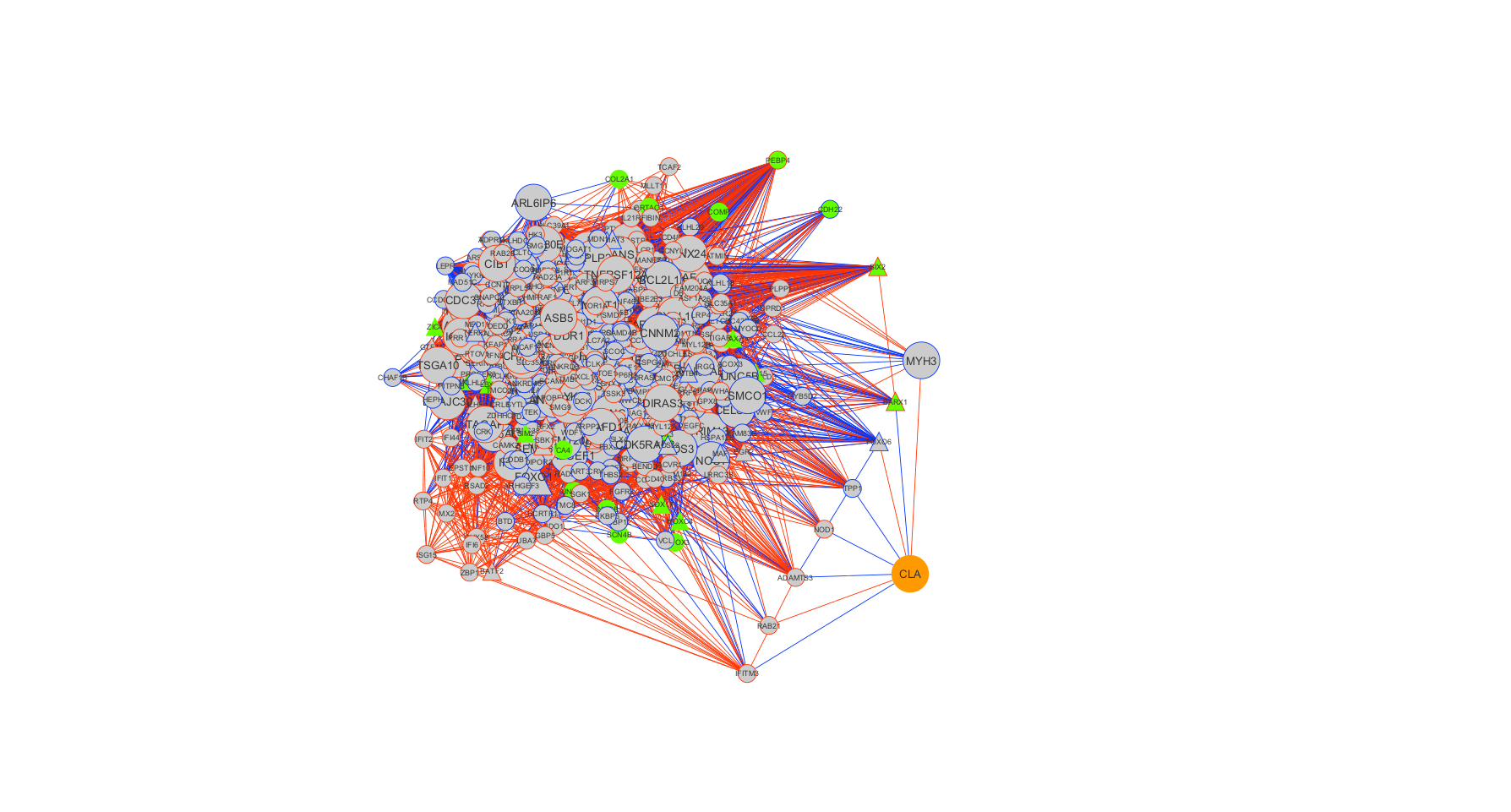
**

**H) OA**

**
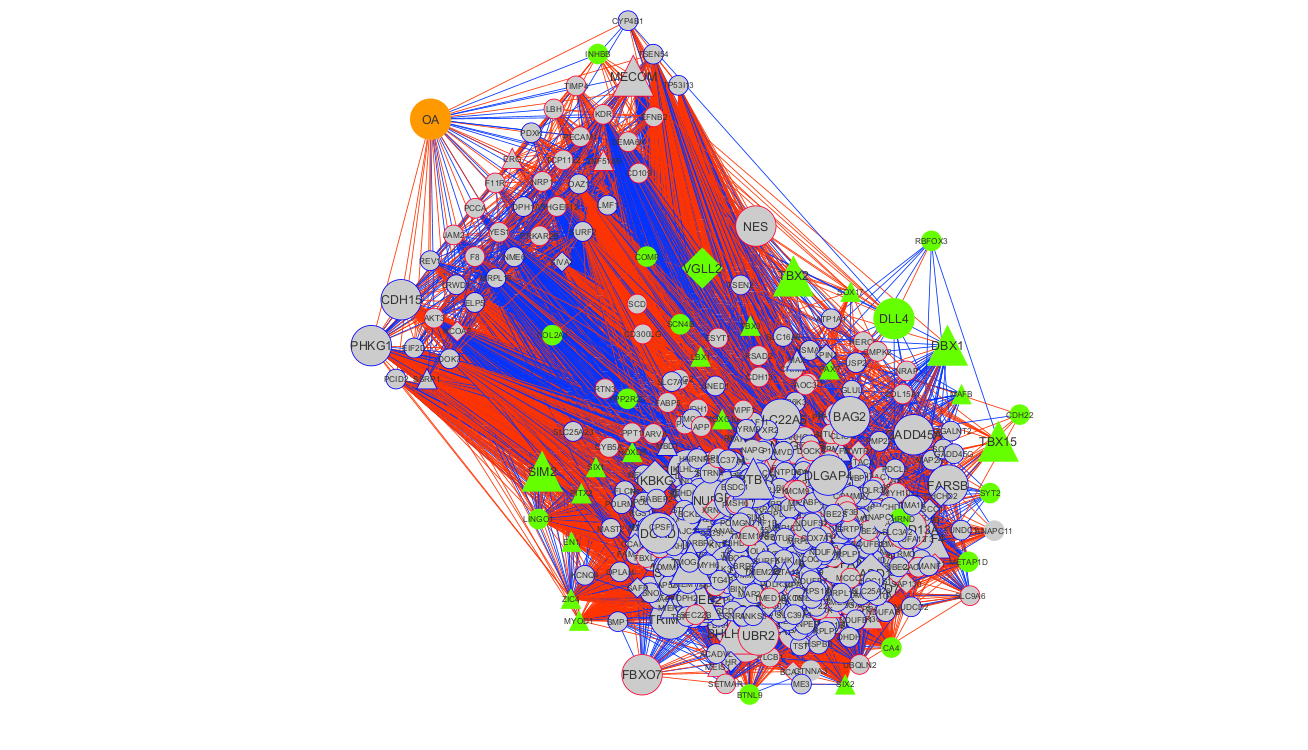
**

1. **PA**

**
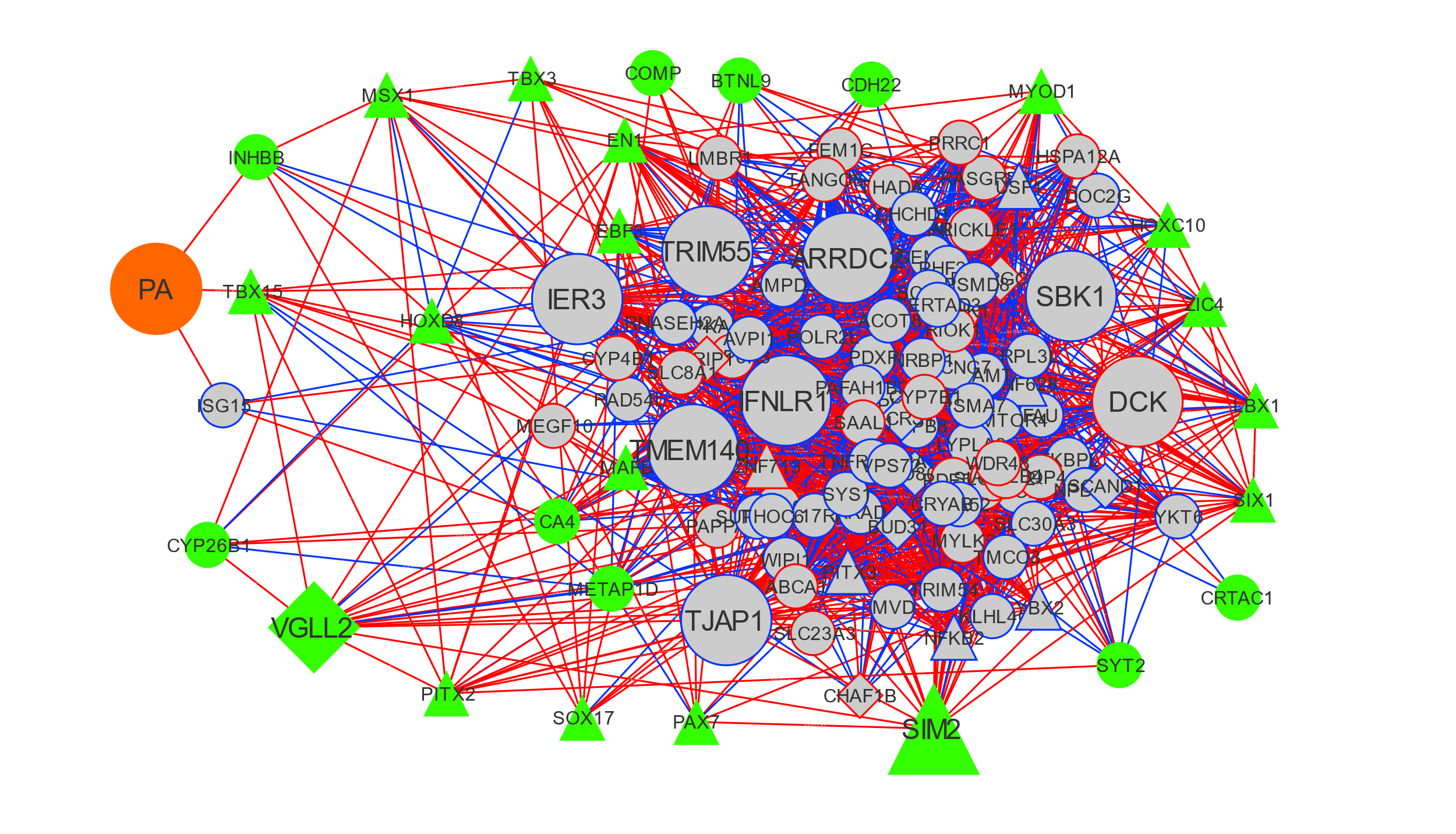
**

**J) EPA**

**
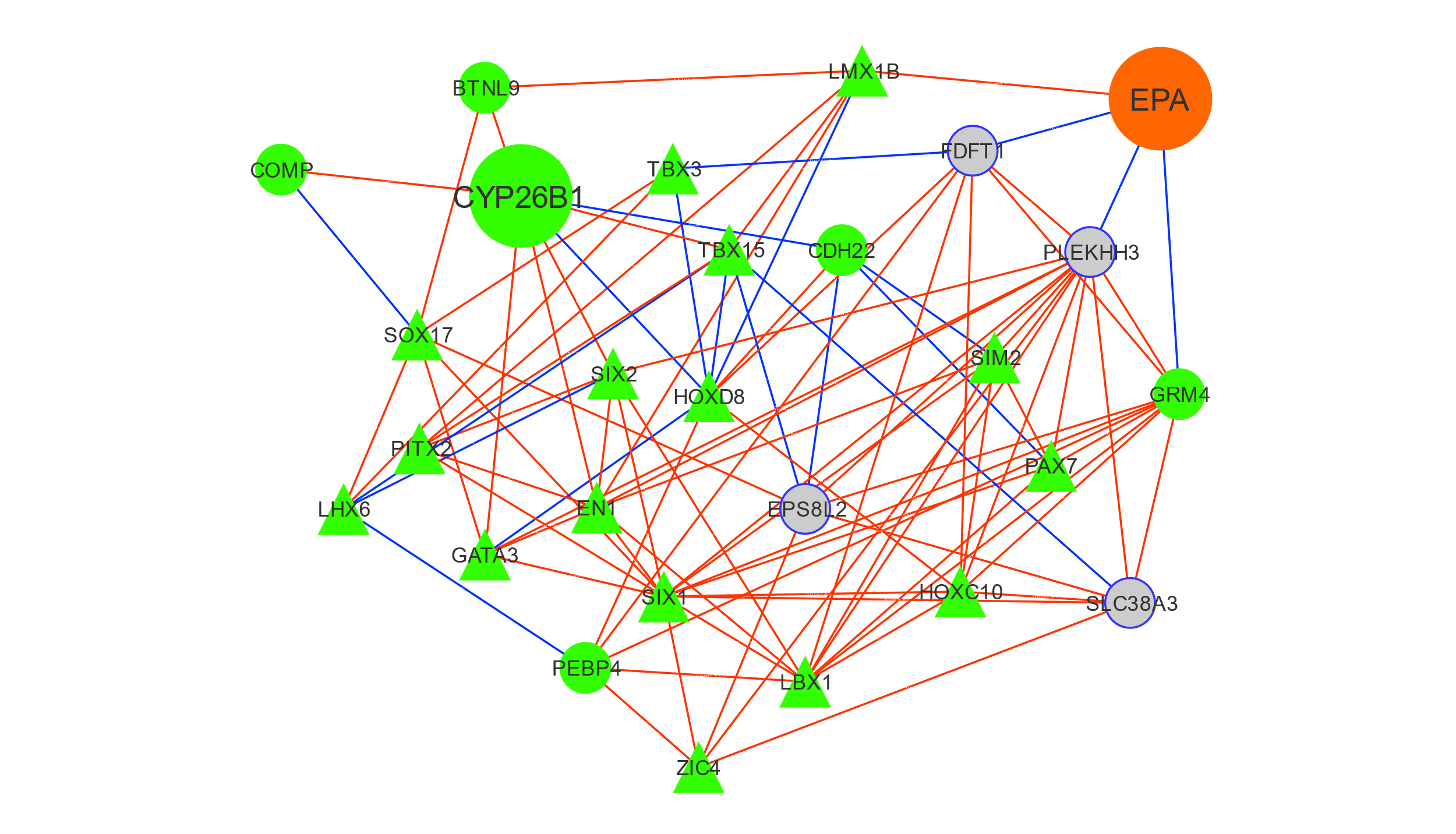
**

**K) DHA**

**
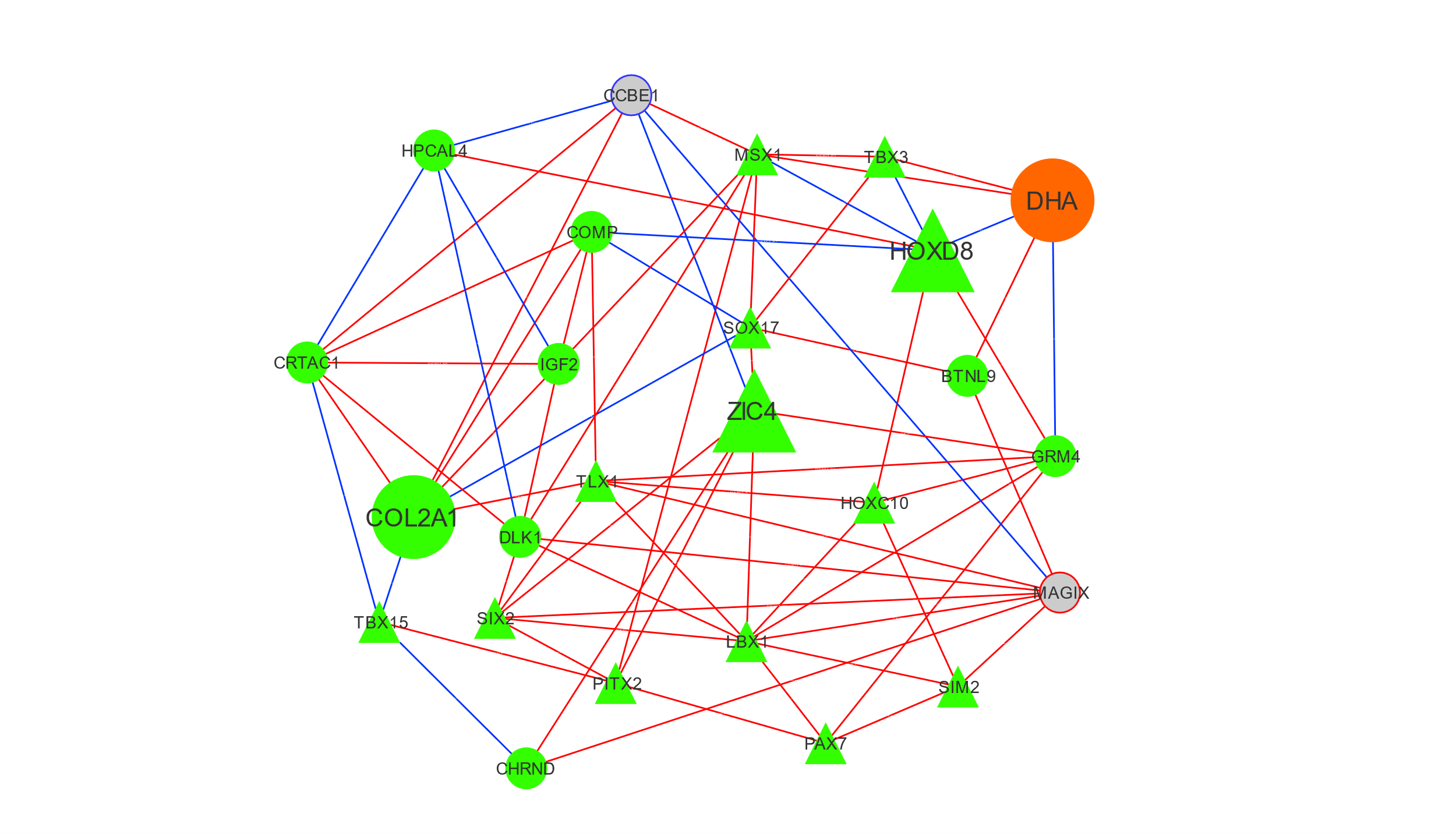
**
